# Optimizing laboratory defined macroprolactin algorithm

**DOI:** 10.11613/BM.2019.020706

**Published:** 2019-06-15

**Authors:** Milica Šostarić, Adriana Bokulić, Domagoj Marijančević, Ivana Zec

**Affiliations:** 1 Faculty of Pharmacy and Biochemistry, University of Zagreb, Zagreb, Croatia; 2 Laboratory of Endocrinology, Clinic of Oncology and Nuclear Medicine, Sestre Milosrdnice University Hospital Center, Zagreb, Croatia

**Keywords:** prolactin, hyperprolactinaemia, macroprolactin, polyethylene glycol

## Abstract

**Introduction:**

Macroprolactinaemia is a well-known analytical problem in diagnostics of hyperprolactinaemia usually detected with polyethylene glycol (PEG) precipitation method. Since there is no harmonization in macroprolactin detection and reporting results, this study proposes and evaluates the usefulness of in-house developed algorithm. The aims were to determine the most suitable way of reporting results after PEG treatment and the possibilities of rationalizing the precipitation procedure.

**Materials and methods:**

This is a retrospective study based on extracted data for 1136 patients. Prolactin concentrations were measured before and after PEG precipitation on Roche cobas e601. Macroprolactinaemia was defined by percentage recovery and post-PEG prolactin concentrations.

**Results:**

Prevalence of macroprolactinaemia using recovery criteria of ≤ 40%, ≤ 60%, and post-PEG prolactin concentrations was 3.3%, 8.8% and 7.8%, respectively. Raising the cut-off value from the upper limit of the manufacturer’s reference interval to 32.9 µg/L does not drastically change detected macroprolactinaemia with recovery criteria. Post-PEG prolactin concentrations showed more than half of the patients with macroprolactinaemia would be overlooked. Regardless of the criteria, a cut-off of 47.0 µg/L would miss most of the macroprolactinaemic patients. Repeated recovery measurements of follow-up patients showed there is a significant difference with mean absolute bias of 9%.

**Conclusions:**

Post-PEG prolactin concentration with corresponding reference interval is the most suitable way of reporting results. All samples with prolactin concentration above the upper limit of the manufacturer’s reference interval should be submitted to PEG precipitation. Follow-up period could be prolonged since the difference between the recoveries of repeated measurements is not clinically significant.

## Introduction

Macroprolactinaemia is a well-known analytical problem in laboratory diagnostics of hyperprolactinaemia. Even though the pathogenesis of this phenomenon is still unclear, it is suggested that macroprolactin complex is a result of genetic predisposition and posttranslational modifications (glycosylation, phosphorylation, deamidation) of the native hormone, which triggers generation of autoantibodies directed at new epitopes (*1*). Because of its high molecular weight, the macroprolactin complex cannot be filtered out easily through the glomeruli resulting in delayed clearance of the bound prolactin and higher concentration of the complex in the blood (*2*). Due to its size and steric hindrance, which prevents binding to prolactin receptors, macroprolactin has insignificant bioactivity. Therefore, most patients with macroprolactinaemia do not exhibit symptoms characteristic for hyperprolactinaemia (*3*). Nevertheless, there have been recorded cases of patients with amenorrhea, galactorrhea, and infertility attributed to intermittent dissociation of macroprolactin complex (*4*).

The main problem in daily practice is the inability of immunoassays to distinguish monomeric prolactin from macroprolactin leading to false diagnosis and unnecessary treatments (*5*). Gel filtration chromatography (GFC) is considered the gold standard for detecting macroprolactin, although it is slow, labour intensive and expensive. Polyethylene glycol (PEG) precipitation is a more suitable method for routine screening since it is cheaper, less time-consuming and has been extensively compared with GFC. The most important limitation of PEG usage is co-precipitation of monomeric prolactin with serum globulins. Studies have shown that even up to 25% of the monomer may be co-precipitated which leads to the false impression of macroprolactin presence (*6*-*8*).

There are two ways of reporting macroprolactinemia presence: as prolactin recovery (%Recovery, %) and prolactin concentration after PEG treatment (post-PEG PRL, µg/L). Mostly used cut-offs for %Recovery are ≤ 40% and ≤ 60% (*9*, *10*). Results reported as %Recovery may be misinterpreted in cases where macroprolactin occurs simultaneously with high concentrations of monomeric prolactin. Nowadays, it is recommended to report results as post-PEG PRL using method-specific post-PEG reference intervals. False or pseudohyperprolactinaemia is defined with post-PEG PRL within post-PEG reference intervals and true hyperprolactinaemia above the upper limit of the post-PEG reference interval (*6*, *11*).

There are no uniform cut-off values for prolactin concentrations that indicate which samples should be treated with PEG. The Endocrine Society guidelines recommend screening for macroprolactin in all asymptomatic patients with elevated prolactin concentrations (*12*). However, every laboratory has its own arbitrary cut-off values depending on the immunoassay method and laboratory PEG precipitation protocol. For example, Whitehead *et al.* routinely screen for macroprolactinaemia only patients with prolactin concentrations above 32.9 µg/L, whereas Suliman *et al.* reported using a cut-off value of 47.0 µg/L (*13*, *14*).

Since there is no harmonization in macroprolactin detection and reporting results, this study proposes and evaluates the usefulness of our in-house developed algorithm.

This study hypothesized there is a difference in macroprolactin detection: (I) according to a different criterion of reporting results; (II) using different cut-off values and (III) in follow-up patients.

The aims were to determine: (I) the most suitable way of reporting results after PEG treatment and (II) the possibility of rationalizing the precipitation procedure (by increasing the cut-off value from the upper limit of the reference interval to 32.9 or 47.0 µg/L and by prolonging follow-up patient period).

## Materials and methods

### Study design

This is a retrospective study with data extracted from the laboratory database for the period from January 2016 to September 2017 in the Laboratory of Endocrinology, Sestre Milosrdnice University Hospital Center (Zagreb, Croatia). Signed informed consent was not necessary since all patients were anonymous and no additional analyses were made on their samples.

**Figure 1:**
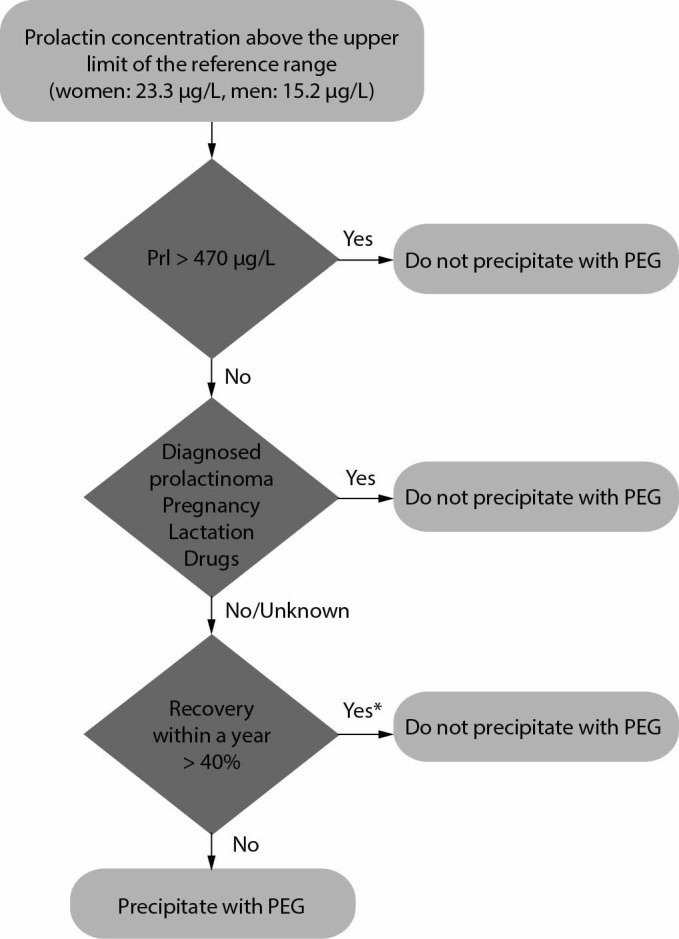
An algorithm with criteria for polyethylene glycol (PEG) precipitation of macroprolactin. *repeated precipitation protocol on samples without fulfilled criteria was carried out by authorized personnel. Prl - prolactin.

All patients with prolactin concentration above the upper reference value defined by the manufacturer (men: 15.2 µg/L; women: 23.3 µg/L) were included in the study according to the in-house algorithm presented in Figure 1. Patients with known causes of hyperprolactinaemia (pregnancy, lactation, prolactinomas) or known use of drugs that increase prolactin concentration (antipsychotics, antidepressants) were excluded from the study. Samples with prolactin concentrations above 470 µg/L were also excluded due to clinical insignificance of macroprolactin presence. Such high concentrations indicate the presence of prolactinoma, therefore even if the macroprolactin is also present, that information will not change the diagnostic treatment of the patient. Post-PEG PRL reference interval (men: 3.0-11.5 µg/L; women: 3.5-17.9 µg/L) was used according to Beltran *et al.* (*6*).

### Subjects

The study population included all out- and inpatients above 18 years of age. The total number of patients was 1136 of which 994 (87.5%) were women and 142 (12.5%) men. Data for repeated measurements were obtained from 71 follow-up patients during the study period.

### Blood sampling and sera preparation

The phlebotomy procedure was done according to the national recommendations for venous blood sampling by the Croatian Society of Medical Biochemistry and Laboratory Medicine (*15*). All blood samples were collected in the morning, at least 2 hours after waking up, using vacuum serum test tubes (Vacuette, Greiner Bio-One GmbH, Kremsmünster, Austria). Prior to sampling, patients were fasting for 8-10 hours and resting for 15 minutes. After 30 minutes clotting time, blood samples were centrifuged at 2200xg for 10 minutes.

### Methods

Prolactin concentrations were measured on Roche cobas e601 analyser (Roche Diagnostics GmbH, Mannheim, Germany) using Elecsys Prolactin II sandwich electrochemiluminescence immunoassay. The method was standardized against the World Health Organization 3rd International Standard 84/500. Total coefficient variations were 3.2% at 7.8 µg/L and 2.8% at 19.8 µg/L.

Sample pre-treatment by PEG precipitation was done according to manufacturer’s recommendations. PEG solution was prepared by dissolving 25 g of PEG 6000 (Merck Schuchardt OHG, Hohenbrunn, Germany) in 100 mL of distilled water and afterward stored at room temperature for 7 days.

An equal volume of serum and 25% PEG solution was mixed for 10 seconds with a vortex mixer and centrifuged at 2200xg for 10 minutes. Prolactin concentration was measured in the supernatant and corrected for the dilution factor (1:2).

The percentage recovery was calculated using initial prolactin (PRL) and post-PEG prolactin concentration (%Recovery = 100 x PRL/post-PEG PRL).

### Statistical analysis

Data distribution normality was tested with the D’Agostino-Pearson test. Wilcoxon rank-sum test is a non-parametric test used for paired measurements with data that does not follow a normal distribution. Hence, it was used to determine the difference between the recoveries of repeated measurements. The level of significance was set at P < 0.05. All statistical analyses were performed using MedCalc (MedCalc Software, version 17.8.6, Ostend, Belgium).

## Results

Data summary statistics are presented in Table 1. D’Agostino-Pearson test showed the data does not follow a normal distribution. Descriptive statistics for age was expressed as median (minimum and maximum). Prolactin concentration before and after PEG treatment was expressed as median (interquartile range).

**Table 1:** Demographic structure and measured results of the studied participants

	**Men** **N = 142**	**Women** **N = 994**	**Total** **N = 1136**
Age, years	48 (18 - 88)	31 (18 - 79)	32 (18 - 88)
PRL, µg/L	25.5 (21.1 - 38.8)	31.8 (26.5 - 46.0)	31.2 (25.9 - 45.4)
Post-PEG PRL, µg/L	20.6 (17.5 - 32.5)	25.4 (21.3 - 37.1)	25.1 (20.8 - 36.2)
%Recovery, %	86 (82 - 90)	84 (79 -87)	84 (79 - 88)
Age is presented as median (min-max), other variables as median and interquartile range. PRL – total prolactin concentration. Post-PEG PRL – prolactin concentration after PEG treatment. %Recovery – prolactin percentage recovery.			

**Figure 2:**
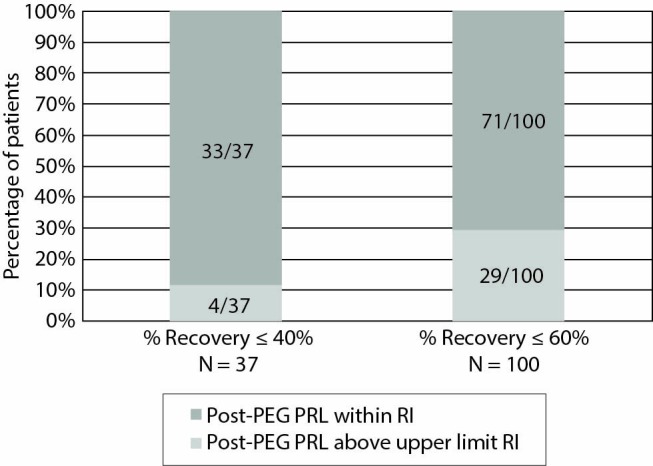
Distribution of patients according to prolactin percentage recovery and post-PEG prolactin values. RI – post-PEG PRL reference interval (men: 3.0-11.5 µg/L; women: 3.5-17.9 µg/L).

Figure 2 depicts the distribution of all patients (N = 1136) according to post-PEG reference intervals within the recovery criteria of ≤ 40% and ≤ 60%. Recovery criterion of ≤ 40% defined 37 patients as macroprolactinaemic. However, post-PEG PRL showed 4 of those patients were still hyperprolactinaemic after the PEG treatment. Recovery criterion of ≤ 60% defined 100 patients as macroprolactinaemic; 29 of them had post-PEG PRL above the upper limit of the post-PEG reference interval (true hyperprolactinaemia).

Macroprolactin detection differed between criteria of reporting results. Recovery criterion of ≤ 40% indicated 3.3% of the total study population were macroprolactinaemic, while the recovery criterion of ≤ 60% showed a higher prevalence of 8.8%. Post-PEG PRL criterion identified 7.8% of patients as macroprolactinaemic.

**Figure 3:**
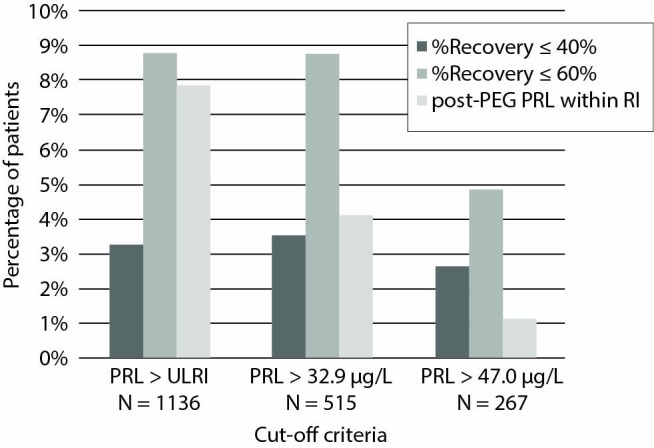
Percentage of patients with macroprolactinaemia depending on the proposed cut-off values and different criteria for determining macroprolactinaemia. ULRI – initial prolactin upper limit of the reference interval (men: 15.2 µg/L; women: 23.3 µg/L). RI – post-PEG PRL reference interval (men: 3.0-11.5 µg/L; women: 3.5-17.9 µg/L).

Figure 3 shows the difference in the prevalence of detected macroprolactinaemia depending on the criteria for submitting samples to PEG treatment and of reporting results. Raising the cut-off value from the upper limit of the manufacturer’s reference interval to 32.9 µg/L does not drastically change the percentage of detected macroprolactinaemia with both recovery criteria (3.3% *vs* 3.5% for recovery of ≤ 40% and 8.8% *vs* 8.7% for recovery of ≤ 60%). However, the post-PEG PRL criterion shows more than half of the patients with macroprolactinaemia would be overlooked (7.8% *vs* 4.1%). The cut-off of 47.0 µg/L is even more restrictive. Regardless of the used criteria, most of the patients with this condition would be missed.

Testing %Recovery data for repeated measurements of follow-up patients (N = 71) showed there is a significant difference (P < 0.001). The mean absolute bias of repeated measurements was 9%.

## Discussion

The prevalence of macroprolactinaemia in general population is around 3.7%. In hyperprolactinaemic population, it varies between 10 and 25% depending on the used methodology and characteristics of the selected study population (*2*, *10*, *16*). Even though GFC is a gold standard for detecting macroprolactin, most of the laboratories use PEG precipitation method with the recovery criterion of ≤ 40% to distinguish false from true hyperprolactinaemia.

The prevalence of macroprolactinaemia in our study population was not as high as in previously reported studies, although it did differ between different criteria for defining macroprolactinaemia. The prevalence of macroprolactinaemia was more than two times higher while using post-PEG reference interval than the recovery criterion of ≤ 40%. The recovery criterion of ≤ 60% detected more macroprolactinaemic patients with the prevalence closer to the post-PEG PRL criterion. The main purpose of the PEG precipitation is to determine whether the bioactive monomeric prolactin concentration is increased (*6*). Regarding definition of macroprolactinaemia, post-PEG reference intervals are more suitable for reporting results.

Currently, according to our in-house protocol, all patients with initial prolactin concentration above the upper limit of the manufacturer’s reference interval undergo PEG treatment. A possible way to rationalize the procedure is by raising the cut-off to previously reported values of 32.9 µg/L and 47.0 µg/L. Both proposed cut-off values did not significantly change the number of detected macroprolactinaemia defined by the recovery ≤ 40%. In the case where the criterion was ≤ 60%, the cut-off of 47.0 µg/L missed almost half of the macroprolactinaemic patients. Lastly, the biggest impact was seen with post-PEG PRL definition of macroprolactinaemia. The 32.9 µg/L cut-off overlooked half of the macroprolactinaemic patients, whereas the 47.0 µg/L cut-off missed even more of them. Since post-PEG PRL is the most reliable way of reporting results, none of the proposed higher cut-off values were acceptable.

Even though we still do not know the real pathogenesis of macroprolactinaemia, it is considered a benign long-lasting condition as Wallace *et al*. have concluded in their study. Additional imaging investigations, dopamine agonist treatments, and prolonged follow-up are not necessary in such cases (*17*). Hattori *et al*. examined whether any new cases of macroprolactinaemia emerged in non-macroprolactinaemic subjects using the recovery criterion of ≤ 40%. Twenty-seven out of 654 subjects were diagnosed with macroprolactinaemia and during the 4-year follow-up period, all of them remained macroprolactinaemic with no significant change in the recovery values. None of the 627 control subjects developed macroprolactinaemia (*18*). Our results showed there is a statistically significant difference in recovery values between repeated measurements. Still, the mean absolute bias of 9% was lower than the intraindividual variation for prolactin by Westgard (23%) concluding the difference is not clinically significant. This indicated it is possible to rationalize our PEG precipitation protocol by prolonging the follow-up period.

The main limitation of this study is its design. Since it is based on retrospective laboratory database extraction, authors did not have insights into patient medical records and were not able to confirm macroprolactinaemia in assessed samples with GFC. This could have been helpful in confirming patients that had macroprolactinaemia along with the increased monomeric form. However, this information does not change the diagnostic treatment of the patient.

In conclusion, the criterion for defining macroprolactinaemia has a big impact on the study results and needs to be considered in data interpretation and comparison with other studies. PEG precipitation is an easy and fast screening method for macroprolactin. Its main purpose should be determining whether the bioactive monomeric prolactin concentration is increased which is why the post-PEG PRL with corresponding reference interval is the most suitable way of reporting results. All samples with prolactin concentration above the upper limit of the manufacturer’s reference interval should be submitted to PEG precipitation. By raising the cut-off to 32.9 µg/L or 47.0 µg/L too many macroprolactinaemic patients would be overlooked. The difference between recoveries of repeated measurements is not clinically significant therefore follow-up period of 1 year could be prolonged.
